# Luteinizing Hormone Receptor Mutation (LHR^N316S^) Causes Abnormal Follicular Development Revealed by Follicle Single-Cell Analysis and CRISPR/Cas9

**DOI:** 10.1007/s12539-024-00646-7

**Published:** 2024-08-16

**Authors:** Chen Zhang, Yongqiang Nie, Bufang Xu, Chunlan Mu, Geng G. Tian, Xiaoyong Li, Weiwei Cheng, Aijun Zhang, Dali Li, Ji Wu

**Affiliations:** 1 Key Laboratory for the Genetics of Developmental and Neuropsychiatric Disorders (Ministry of Education), Bio-X Institutes, Shanghai Jiao Tong University, Shanghai, 200240 China; 2 Department of Obstetrics and Gynecology, Ruijin Hospital, Shanghai Jiao Tong University School of Medicine, Shanghai, 200025 China; 3 School of Basic Medical Sciences, Key Laboratory of Fertility Preservation and Maintenance of Ministry of Education, Ningxia Medical University, Yinchuan, 750004 China; 4 International Peace Maternity and Child Health Hospital, Shanghai Jiao Tong University School of Medicine, Shanghai, 200030 China; 5 Shanghai Key Laboratory of Regulatory Biology, Institute of Biomedical Sciences and School of Life Sciences, East China Normal University, Shanghai, 200241 China; 6 Department of Hematology, Tangdu Hospital, Xi’an, 710032 China

**Keywords:** Oocytes, Granulosa cells, Follicle, LHR^N316S^, Single cell RNA-seq, Progesterone

## Abstract

Abnormal interaction between granulosa cells and oocytes causes disordered development of ovarian follicles. However, the interactions between oocytes and cumulus granulosa cells (CGs), oocytes and mural granulosa cells (MGs), and CGs and MGs remain to be fully explored. Using single-cell RNA-sequencing (scRNA-seq), we determined the transcriptional profiles of oocytes, CGs and MGs in antral follicles. Analysis of scRNA-seq data revealed that CGs may regulate follicular development through the BMP15-KITL-KIT-PI3K-ARF6 pathway with elevated expression of luteinizing hormone receptor (LHR). Because internalization of the LHR is regulated by *Arf6*, we constructed LHR^N316S^ mice by CRISPR/Cas9 to further explore mechanisms of follicular development and novel treatment strategies for female infertility. Ovaries of LHR^N316S^ mice exhibited reduced numbers of corpora lutea and ovulation. The LHR^N316S^ mice had a reduced rate of oocyte maturation in vitro and decreased serum progesterone levels. Mating LHR^N316S^ female mice with ICR wild type male mice revealed that the infertility rate of LHR^N316S^ mice was 21.4% (3/14). Litter sizes from LHR^N316S^ mice were smaller than those from control wild type female mice. The oocytes from LHR^N316S^ mice had an increased rate of maturation in vitro after progesterone administration in vitro. Furthermore, progesterone treated LHR^N316S^ mice produced offspring numbers per litter equivalent to WT mice. These findings provide key insights into cellular interactions in ovarian follicles and provide important clues for infertility treatment.

**Graphical Abstract:**

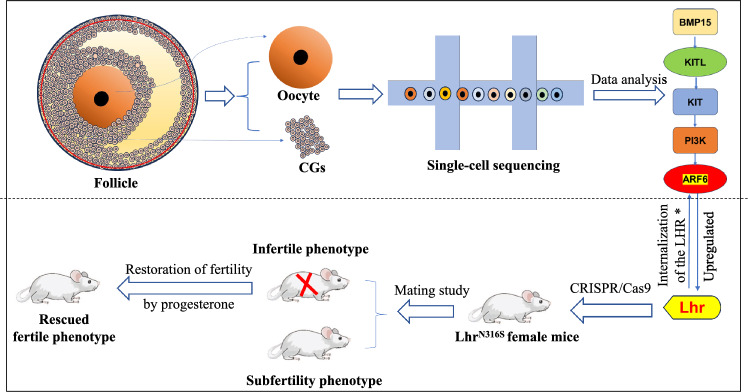

**Supplementary Information:**

The online version contains supplementary material available at 10.1007/s12539-024-00646-7.

## Introduction

In mammalian reproduction, the ovarian follicle comprises a central oocyte with surrounding granulosa cells (GCs) and theca cells [1, 2]. The GCs regulate oocyte cortex dynamics and germinal vesicle (GV) position, anchor and spindle during oocyte maturation [3–5]. Bidirectional communication between oocytes and GCs contributes to oocyte development [1, 2, 6, 7], and abnormal interaction of this system causes disordered follicle development [8–10]. The GCs from antral follicles include both cumulus granulosa cells (CGs) and mural granulosa cells (MGs). MGs are located in the outer layer of GCs in antral follicles, and CGs closely encircle the oocyte. The two types of GCs have different responsiveness to extracellular signals, and different roles and fates in follicular development [11–14]. The MGs produce estrogen, which triggers the pituitary to secrete luteinizing hormone (LH), resulting in the promotion of oocyte maturation, cumulus expansion and ovulation. The CGs promote oocyte growth and maturation [15–17]. In a previous study, interactive regulation between oocytes and GCs during follicular development was shown at the transcriptional level via RNA-sequencing (RNA-seq) analysis [18]. However, the interactions between oocytes and CGs, oocytes and MGs, as well as CGs and MGs remain to be fully explored. Single cell RNA-seq (scRNA-seq) of the antral follicle would provide a targeted means to analyze these cellular interactions.

ADP-ribosylation factor 6 (*Arf6*) is a member of the ARF family of small GTPases that regulates multiple cellular events, cycling between active GTP and inactive GDP-bound forms [19]. Cavenagh et al. reported high expression levels of *Arf6* in the human ovary [20]. Furthermore, it was reported that *Arf6* was necessary for oocyte maturation [21]. Interestingly, *Arf6* regulates the internalization of the luteinizing hormone receptor (LHR) and other G protein-coupled receptors (GPCRs) [22]. Previous studies showed that disruption of *Arf6* in mice was fatal to embryos [23]. Therefore, *Arf6* knockout mice do not provide a feasible model to study the effects of *Arf6* on antral follicular development.

The LHR is essential for ovulation, progesterone production [24] and fertility [25]. In the ovary, LHR is present in theca cells, GCs, stromal cells and luteinized cells. Mutations and polymorphisms of LHR have been shown to cause infertility, subfertility and poor outcomes of fertility treatment [26]. In humans, the LHR^N312S^ (rs2293275) variant is one of the most frequently studied polymorphic sites [27], LHCGR N312 may also be helpful for predicting reproductive outcomes in ART [28–30]. However, the underlying mechanism of the action of LHR^N312S^ impacting female fertility is unknown, and there is no clear strategy for the treatment of female infertility caused by LHR^N312S^. In mice, the LHR^N316S^ site corresponds to the LHR^N312S^ site in humans and may provide a valuable model to study the potential mechanisms of LHR^N316S^ actions on female fertility.

The current study detected the transcriptional profiles of oocytes, CGs and MGs in antral follicles by scRNA-seq and analyzed the interactions between these profiles. Then, we generated LHR^N316S^ mice by CRISPR/Cas9 technology to explore the mechanisms of follicular development and a novel treatment strategy for female infertility.

## Experiments

### Animals

All procedures regarding to the animal experiment were approved by the Institutional Animal Care and Use Committee of Shanghai, and were conducted in line with the National Research Council Guide for Care and Use of Laboratory Animals. C57BL/6J and ICR mice were supplied by the company of Shanghai SLAC laboratory animal, China.

### Isolation of Single Cells from Individual Antral Follicles

Female C57BL/6J mice at 6 weeks old were anesthetized and euthanized according to the experimental guidelines of animals in Shanghai Jiao Tong University. The ovaries were harvested and put into pre-cold phosphate-buffered saline (PBS). After that, mice ovaries were washed with pre-cold PBS and separated into several pieces under a stereomicroscope using a 29 G1/2 needle attached to an insulin syringe. Then, individual antral follicles were picked up with a mouth pipette and washed with cold PBS at least three times to remove any contaminating small cells. For collection of single cells, an individual antral follicle was transferred into a drop of PBS and gently punctured by a 29 G1/2 needle. Clumps of MGs and COCs were transferred into different drops of PBS. After several washes in PBS, MGs were scattered and a single cell was selected under a manipulation system. The COC was washed thoroughly in PBS with 1% hyaluronidase to separate CGs and the oocyte. A single oocyte or single CG was selected with a mouth pipette or manipulation system. Two oocytes were obtained from other follicles of similar size in repeated experiments.

### cDNA Preparation and Library Preparation for Next Generation Sequencing

An individual cell was selected as described above and transferred into commercialized lysis buffer. The cDNA of single cell was reverse-transcribed with cell lysis using a SMARTer^®^ Ultra™ Low Input RNA for Sequencing kit (Clontech Laboratories, Inc., Mountain View, CA) following the manuals. Briefly, RNA with polyA was reverse transcribed to cDNA using enzyme and primers for reverse transcription in the kit mentioned above. cDNA was amplified with 18 PCR cycles and purified step by step following the manuals. The amount and quality of purified cDNA libraries were determined by Agilent 2100. Fity nanogram tagmentation cDNA was prepared using Covaris S2 and a commercialized kit named NEBNext^®^ Ultra™ for Illumina platform. The tagmentation cDNA was ligated with adapters after terminus repairing and dA tailing. Then, the cDNA with adapters was amplified with 12–15 PCR cycles. Paired-end sequencing was performed on a HiSeq 2500.

### Read Mapping and Quantification of Gene Expression Levels

Raw data of each sample in FASTQ files were first processed into clean data by filtering the low-quality reads, reads with adapter and ploy-N. The quality of clean data was assessed based on the sequencing quality score of 20, of 30 and GC content. All the clean data was qualified with high quality and was used for following analysis.

Reference genome of mice and the annotation files were downloaded from the genomic libraries. Bowtie2 with version of v2.2.5 was used for building a reference genomic index. TopHat v2.0.14 was used for aligning the paired-end clean reads to the reference genome. The number of read mapping to each gene was counted by HTSeq v0.6.1. Then, the FPKM of each gene was determined in all the samples. A flow chart for the data analysis was shown in Fig. S1. A list of the package and software to analyze the data was shown in Supplementary Table S1.

### Weighted Gene Co-expression Network Analysis (WGCNA)

Genes expressed at FPKM value ≥ 0.1 in any of the samples were used for constructing a signed network. Briefly, the correlation between the expression values of gene *i* and the eigengene of module *q* was defined as MMq(*i*) = cor[*x*(*i*), *E*^*q*^], where *x*(*i*) is the expression profile of gene *i* and *E*^*q*^ is the eigengene of module *q* [31]. Modules were clustered using the method of dynamic hybrid cut.

Gene co-expression networks, which could assess the intensity of gene interactions, were built according to the expression levels of genes (FPKM). The network was constructed based on the significant Pearson correlation pairs of genes [32]. Degree centrality, which determines relative importance of gene node, was defined as the number of links one node has to the others within a network [33, 34]. *K*-cores were used to find the very important subgraphs in the co-expression networks.

### Gene Ontology Term and Pathway Enrichment Analysis

The main biological domain of the differential expression genes was analyzed by GO analysis, based on the Wey functional classification of NCBI [35]. The significant enrichment of GO terms was scored by hypergeometric test. False discovery rates were estimated using either Bonferonni or Benjamini–Hochberg procedures.

The significant biological functions of differentially expressed genes were analyzed by pathway enrichment analysis, based on the Fisher exact test. The *P* adjusted values were calculated by the algorithm of BH FDR [36]. The FDR values of all the reported pathway categories were less than 0.05.

### Single-Cell Quantitative PCR

Single cell qPCR was applied for independent of the RNA-seq analyzed embryos, another four CGs, six MGs and five oocytes. Each single cell was snap frozen and stored at − 80 °C. HiScript II Q RT SuperMix for qPCR (+gDNA wiper) kit (Vazyme, China) was used for cell reverse transcription. Fast Start Universal SYBR Green Master Mix kits (Roche, Germany) was applied for primer-specific amplification with ABI PRISM 7500 system (Applied Biosystems, USA). The primers are shown in Supplementary Table S12.

### Construction of LHR^N316S^ Mutant Mice

Methods for generation of the point mutation in mice were described previously with modifications [37]. Briefly, Tris–EDTA buffer containing 50 ng/μg Cas9 mRNA, 50 ng/μL sgRNA1, 50 ng/μg sgRNA2 and 100 ng/μL ssODN donor was injected into the pronuclei of one-cell stage mouse embryos using an Eppendorf transfer Man NK2 micromanipulator. Injected embryos were transferred into KSOM medium and cultured overnight before transfer into pseudopregnant C57BL/6 female mice. Offspring tail-tip biopsies were subjected to PCR amplification using a primer pair specific for *Lhr* to amplify an *Lhr* fragment, and LHR^N316S^ mutant mice were identified by genomic sequencing. C57BL/6 LHR^N316S^ mice were mated with ICR wild-type mice to obtain C57BL/6 × ICR LHR^N316S^ mice.

### Hormone Assay

Blood was taken from the tail vein of mice. The levels of serum progesterone and serum estradiol were detected by the Ruijin Hospital, Shanghai Jiao Tong University School of Medicine (Shanghai, China).

### Fertility Assessment and Progesterone Therapy

Adult LHR^N316S^ female mice were injected intraperitoneally with 10 IU PMSG (Sansheng, China) and 10 IU human chorionic gonadotropin (Sansheng, China) 48 h apart. After approximately 14 h, metaphase II oocytes were collected from oviducts. Wild-type females with the same age as the LHR^N316S^ mutant females were used as controls.

Mouse COCs isolated from 10 IU PMSG-primed mice were cultured in IVG medium: α-MEM supplemented with 5% FBS (Sigma), 150 μmol/L ascorbic acid, 1 × glutamax, 1 × penicillin/streptomycin, 100 μmol/L 2-mercaptoethanol, 55 μg/mL sodium pyruvate (NacalaiTesque), 0.1 IU/mL follicle-stimulating hormone (Follistim; MSD), 15 ng/mL BMP15 and 15 ng/mL growth differentiation factor 9 (R&D Systems), in the presence or absence of 10 μM progesterone. Maturation and cumulus expansion were detected at the end of 36 h of culture.

Six- to 12-week-old LHR^N316S^ mutant female mice were selected to a continuous mating study. Wild-type female mice acted as control. Two female mice mated with one 8- to 10-week-old fertile wild-type male mouse. Then, the numbers of offspring per litter were recorded.

Fourteen LHR^N316S^ female mice were mated with fertile WT males, and 14 WT females of the same age were mated with fertile WT males as a control group. Two months after mating, female mice not pregnant received progesterone treatment were injected subcutaneously with 50 mg/kg progesterone (progesterone treatment group). The mice that were still not pregnant were treated with a second dose of 50 mg/kg progesterone, and the number of post-generation was counted.

### Histology

Mouse ovaries were fixed in 4% paraformaldehyde at 4 °C for 24 h. Then, the ovaries were encapsulated in paraffin and sectioned at 6 µm. Then, slides were stained with H&E. Images were obtained with microscope (Leica, DM2500) and digital camera (Leica, DFC550).

### Statistics

The results were presented as mean ± standard error of the mean (SEM). Means were compared using two-tailed, unpaired Student’s *t* tests by SPSS software. *P* < 0.05 was considered as statistically significant. All experiments were repeated three times.

## Results

### Single-Cell Transcriptional Profiles of Oocytes, Cumulus Granulosa Cells and Mural Granulosa Cells in Antral Follicles

**Fig. 1:**
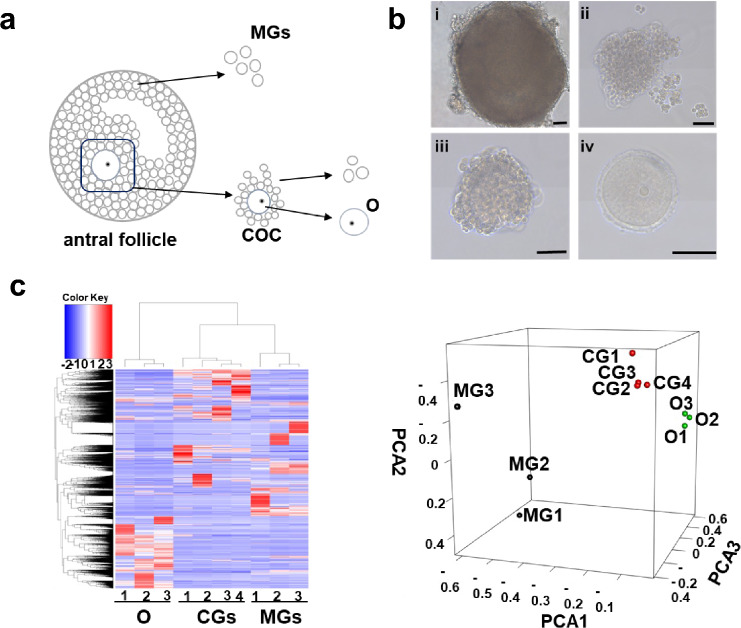
Transcriptional profiles of single cells from the three types of cells examined in an antral follicle. **a** Schematic diagram of isolating individual cell types from a single antral follicle. **b** Morphology of a freshly isolated mouse antral follicle (i), mural granulosa cell (MG) (ii), cumulus-oocyte-complex (COC) (iii), and oocyte (O) (iv). **c** Unsupervised clustering of the transcriptome of all the samples. **d** Principal component analysis (PCA) of single cell expression patterns from the three type cells. Bar 50 μm

To investigate the interactions between oocytes and CGs, oocytes and MGs, and CGs and MGs, we detected cellular transcriptional profiles by scRNA-seq. Single cell types (oocytes, MGs and CGs) were isolated from antral follicles (Fig. 1a and b). Approximately 300 million sequencing reads derived from 10 single cells were mapped in this study. All genes expressed in these cells are listed in Supplementary Table S2. To ensure the reliability of scRNA-seq data, a *Q*-score higher than 30 (error rate < 0.1%) was acquired for each replication. To determine whether these gene expression profiles were correlated with different cell types, scRNA-seq data of all samples was analyzed by unsupervised hierarchical clustering. The cells that clustered together naturally were at the same cell type in all samples (Fig. 1c). These results were supported by principal-component analysis (Fig. 1d). Transcriptional profiles were obtained for oocytes, CGs and MGs in antral follicles.

### Analysis of the Signals for the Communication Between Oocytes and Granulosa Cells

**Fig. 2:**
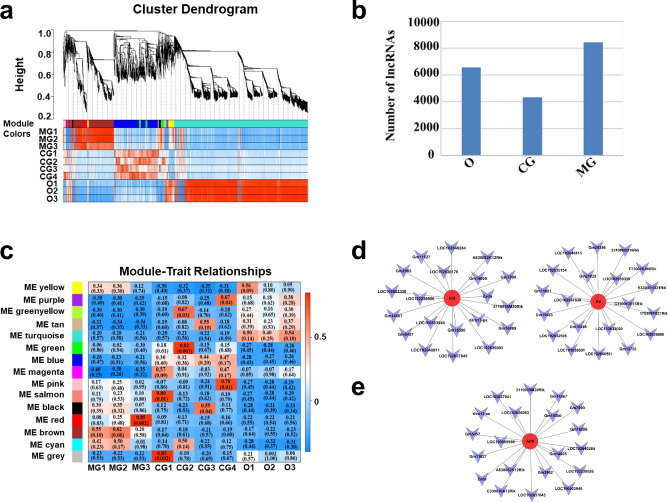
Gene-network modules established by weighted correlation network analysis (WGCNA), and oocyte polarity established gene-related long non-coding RNA (lncRNA). **a** WGCNA dendrogram indicating the expression of different gene modules in all 10 single-cell samples. **b** Module trait relationship followed by *P* values in parentheses between modules and different samples. **c** The number of lncRNAs detected. **d** and **e** LncRNAs correlated with genes *Kitl*, *Kit* and *Arf6*. Genes with different colored cycles mean different ranks in the network (ranks from higher to lower are red, green, yellow, dark blue and light blue). *CG* cumulus granulosa cells, *O* oocytes, *MG* mural granulosa cells

To study the regulation of oocytes by GCs, scRNA-seq data (fragments per kilobase per million mapped fragments, FPKM ≥ 0.1) were subjected to weighted correlation network analysis (WGCNA), and 15 gene-network modules were established (Fig. 2a and b, and Supplementary Table S3). Gene Ontology (GO) analysis of these modules revealed several key biological processes related to oocyte maturation. These biological processes were mainly enriched in the brown and turquoise modules shown in Supplementary Tables S4 and S5. Genes that functioned in the processes of oocyte maturation and follicle development were preferentially selected from these two modules. Approximately 300 selected genes were subjected to the construction of a co-expression network (Fig. S2 and Supplementary Table S6). In the analysis of the gene co-expression network, the expression of differential genes reflects the intergenic synergistic relationships. To identify the tightness of the co-expressed relationships among these genes, *K*-core values of genes were calculated (Supplementary Table S11) to focus on genes with *K*-core values ≥ 17 (Top2).

**Fig. 3:**
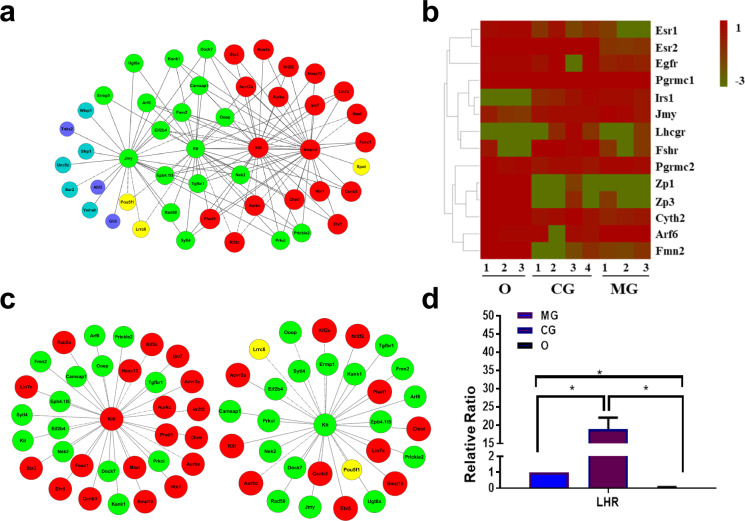
Gene-network and gene expression differences. **a** Genes co-expressed with Kit ligand (Kitl) and Kit in the network. **b** Genes co-expressed with Kitl in the network. Genes co-expressed with Kit in the network. **c** The gene expression of related genes in the three types of cells. **d** The validation of *Lhr* expression by qRT-PCR*.* *O*-oocytes, *CG*-cumulus granulosa cells, *MG*-mural granulosa cells. *P < 0.05, *n.s.* no significant difference

The *Kitl*-*Kit*, which is a canonical signal pathway involved in the communication between oocytes and GCs [38–43], was identified within this co-expression network (Fig. 3a). Nineteen genes that co-expressed with *Kitl* are shown in Fig. 3b. Upstream of this pathway, *Bmp15* was co-expressed with *Kitl* (Fig. 3b). It has been reported that bone morphogenetic protein 15 (*Bmp15*) is secreted from oocytes and regulates the expression of *Kitl* in CGs [1]. Thirty genes were co-expressed with *Kit* (Fig. 3b). All the genes co-expressed with *Kit* and *Kitl* were represented in a network shown in Fig. 3a. Among these co-expressed genes, *Arf6* was selected as a candidate that functioned downstream of the *Kitl*-*Kit* signal pathway.

*Arf6* was highly expressed in the GV oocyte samples (average FPKM = 13.63, Supplementary Table S2 shows FPKM values of all genes). It was reported that cytohesin 2 activates *Arf6* in a phosphoinositide 3-kinase (PI3K)-dependent manner during pre-adipocyte migration [44]. Interestingly, cytohesin 2 was also highly expressed in oocytes in this study (average FPKM = 18.88, Supplementary Table S2). It has been reported that the intra-oocyte PI3K pathway was activated by KITL-KIT in growing oocytes, inducing multiple effects, such as promoting the secretion of BMP15 [40, 45]. Therefore, we predicted that GCs may regulate oocyte maturation and follicular development through the BMP15-KITL-KIT-PI3K-ARF6 pathway.

### Identification of Long Non-Coding RNAs Correlated with Genes Involved in Oocyte Maturation Based on WGCNA

Long non-coding RNAs (IncRNAs) are typically over 200 nucleotides in length and involved in diverse biological processes. The total numbers of lncRNAs detected in oocytes, CGs and MGs were 6545, 4357 and 8444, respectively (Fig. 2c). To identify possible lncRNAs involved in follicular development, module membership assignment and the weight of each gene from brown were calculated in WGCNA (Supplementary Tables S7 and S8). The top 20 lncRNAs correlated with *Bmp15, Kitl, Kit* and *Arf6* were identified, as shown in Figs. 2d, e and S3, and Supplementary Tables S9 and S10. These lncRNAs may regulate follicular development through molecular members involved in the BMP15-KITL-KIT-PI3K-ARF6 pathway.

### Generation of LHR^N316S^ Mice

**Fig. 4:**
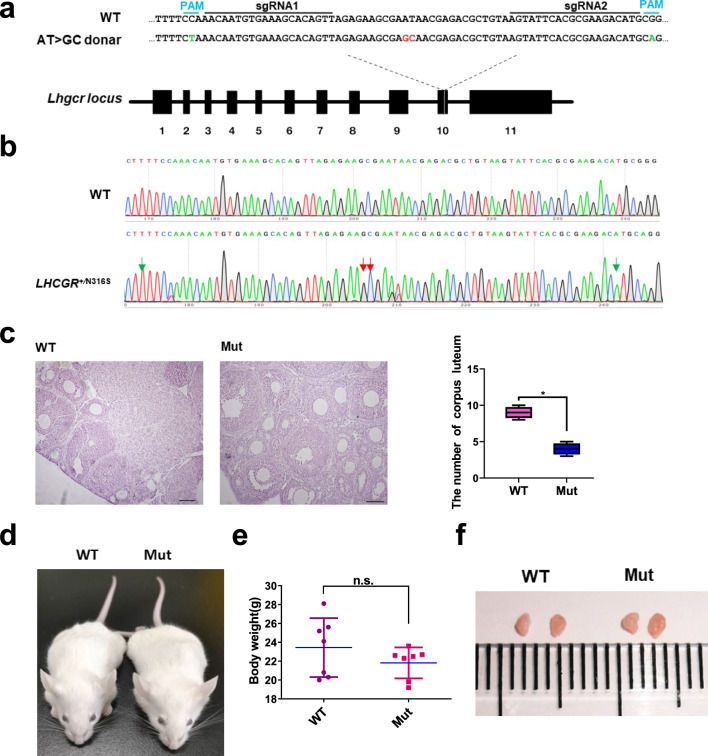
The generation of luteinizing hormone receptor (LHR)^N316S^ mice. **a** Schematic view of the strategy used to generate the N316S point mutation (LHR^N316S^). Base pair substitutions for N316S are labeled in red. Silent mutations to prevent cleavage of the precisely mutated alleles are labeled in green. **b** Sanger sequencing chromatogram of genomic DNA from a wildtype (WT) mouse and the LHR^N316S^ F0 founder. Red arrows indicate the base pair substitutions for the N316S point mutation. Green arrows indicate the silent mutations. **c** Morphological comparison of ovaries from LHR^N316S^ and control female mice (left). The number of corpora lutea in different groups (right). Bar 100 μm. **d** The WT and LHR^N316S^ C57BL/6 × ICR mice 8 weeks after birth. **e** The mean body weight of LHR^N316S^ female mice was indistinguishable from that of controls (female littermates of LHR^N316S^ female mice). **f** Images of whole ovaries from 8-week-old LHR^N316S^ and control mice. *P < 0.05, *n.s.*-no significant difference

It is known that *Arf6* is necessary for antral follicular development [46], but the *Arf6* knockout mouse exhibits embryonic lethality [23]. It was reported that *Arf6* regulates the internalization of LHR and other GPCRs [22]. Moreover, LHR directly activates the Arf-nucleotide binding site opener protein, which in turn activates *Arf6* by promoting GTP/GDP exchange [47]. Consistent with the literature, the present study found that *Arf6* was highly expressed in the three kinds of cells examined (Fig. 3c) and the *Lhr* was highly expressed in CGs (Figs. 3d and S4). These findings suggested that the *Lhr* may act downstream of *Arf6* in follicular development. In humans, the N312S mutation of LHR has been shown to potentially inhibit oocyte maturation [27, 48]. This mutation corresponds to the murine LHR mutation at N316S (LHR N316S). To research the role of LHR^N316S^ in follicular development in mice, we generated the LHR^N316S^ mouse model using CRISPR/Cas9 technology (Fig. 4a, blue: silence mutation; red: N to S mutation). Genomic sequencing was used to confirm the successful generation of LHR^N316S^ mice, and heterozygous LHR^N316S^ mice were selected for further study (Figs. 4b and S5).

### LHR^N316S^ Causes Subfertility in Female Mice

**Fig. 5:**
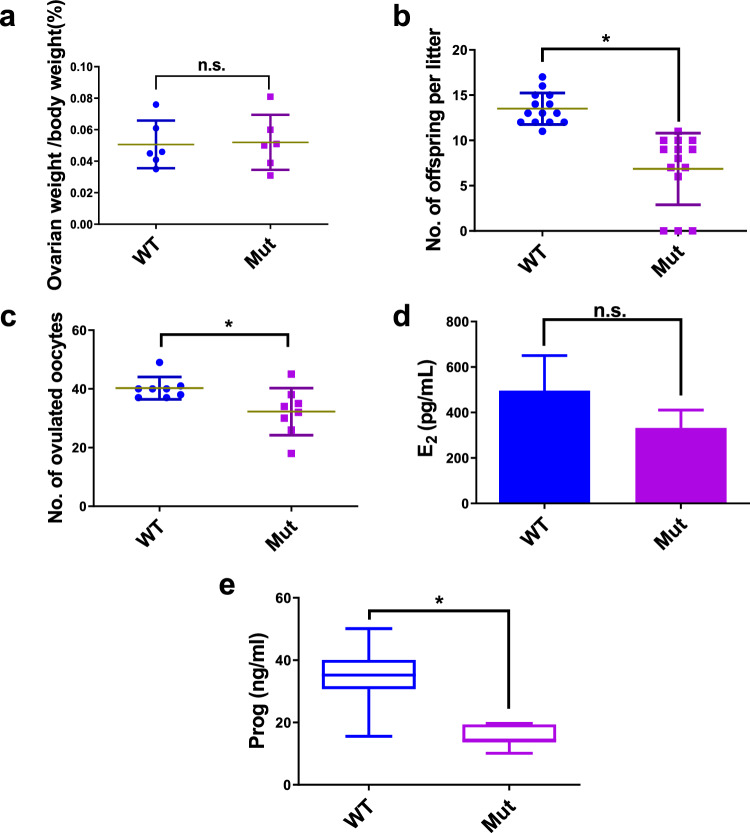
Low fertility of luteinizing hormone receptor (LHR)^N316S^ adult female mice. **a** The relative ovarian weight of LHR^N316S^ mice was indistinguishable from wild type (WT) mice. **b** Litter sizes from C57BL/6 × ICR (*n* = 14) LHR^N316S^ female mice mated with ICR WT male mice were smaller than those from C57BL/6 × ICR (*n* = 14) WT female mice mated with ICR WT male mice. **c** The mean number of ovulated oocytes per female mouse after hormonal stimulation. **d** Estradiol (E2) levels in WT and LHR^N316S^ mice. **e** Progesterone (Prog) homone levels in WT and LHR^N316S^ mice. LHR^N316S^ and WT adult female mice were used at the same ages. Data shown as mean ± SEM, *n* ≥ 6 mice per group. *P < 0.05, *n.s.*-no significant differences

In present study, the number of corpora lutea was significantly lower in the LHR^N316S^ group compared with that in the control group (*P* < 0.05, Fig. 4c). However, there were no differences in body weight (Fig. 4d and e), ovarian morphology (Fig. 4f) and the ovary-to-body weight ratio (Fig. 5a) of 6-week-old LHR^N316S^ vs control mice (*P* > 0.05). To investigate fertility, 6- to 10-week-old LHR^N316S^ and control female mice were mated naturally and the number of offspring per litter was recorded. Although most LHR^N316S^ females (11/14) produced offspring after mating with fertile males, the number of offspring per litter was lower than the control group (*P* < 0.05), indicating subfertility (Fig. 5b). Moreover, the number of ovulated oocytes induced by pregnant mare serum gonadotropin (PMSG) and human chorionic gonadotropin was lower in LHR^N316S^ compared with that in control females (Fig. 5c). These results suggested that the LHR^N316S^ female mice were subfertile.

Because the LHR^N316S^ female mice were subfertile, potential hormonal changes caused by LHR^N316S^ were examined in 6-week-old female mice. Compared with the findings in wild type (WT) mice, there were no significant differences in serum estradiol levels (Fig. 5d), but significantly lower serum progesterone levels in 6-week-old LHR^N316S^ mice (Fig. 5e), suggesting that the reduced oocyte maturation caused by LHR^N316S^ may be due to decreased progesterone.

### Progesterone Improves Oocyte Maturation and Reproduction of LHR^N316S^ Mice

**Fig. 6:**
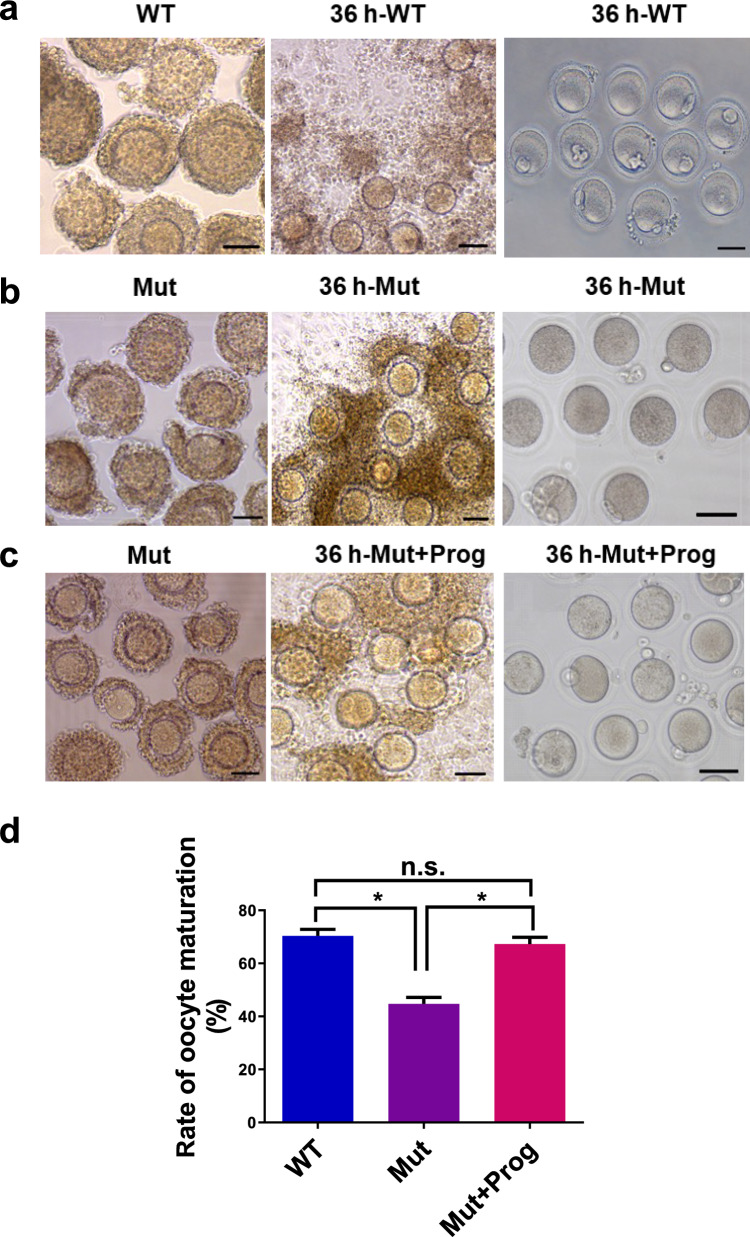
Effect of progesterone on cumulus expansion and oocyte maturation. Cumulus oocyte complexes (COCs) were isolated from mice (48 h after pregnant mare serum gonadotropin stimulation) by puncturing large antral follicles and then cultured in IVM medium supplemented with or without 10 μmol/L progesterone for 36 h. **a** COCs from wild type (WT) mice were incubated in IVM medium. **b** COCs of luteinizing hormone receptor (LHR)^N316S^ mice were incubated in IVM medium. **c** COCs from LHR^N316S^ mice were incubated in IVM medium with 10 μmol/L progesterone. **d** The oocyte maturation rate of oocytes from LHR^N316S^ mice incubated in IVM medium with or without progesterone. LHR^N316S^ and WT female mice were used at the same ages. Bar 50 μm. *P < 0.05, *n.s.*-no significant differences

The present study examined progesterone actions on oocyte maturation in mice using in vitro and in vivo models. After cumulus-oocyte complexes (COCs) isolated from PMSG-primed mice were cultured for in vitro maturation (IVM) for 36 h, the number of mature oocytes was lower from LHR^N316S^ mice (44%) than from WT mice (70%, *P* < 0.05) (Fig. 6a, b and d). The oocyte maturation rate of progesterone-treated COCs from LHR^N316S^ mice was higher (67%) than untreated COCs from LHR^N316S^ mice (44%, *P* < 0.05) and was equivalent to the COCs from WT mice (*P* > 0.05) (Fig. 6c and d). These results suggested that progesterone can significantly promote oocyte maturation in vitro.

To examine whether progesterone can improve oocyte maturation in vivo, 14 LHR^N316S^ and 14 WT female mice were mated with WT males. All WT mice became pregnant. However, three LHR^N316S^ mice did not become pregnant, and the infertility rate of mutant mice was 21.4% (3/14). Female LHR^N316S^ mice treated with progesterone in vivo produced offspring with normal litter sizes (Table 1).

**Table 1:** Progesterone treatment of infertility mice

	Mice treatment	Total number of females	Pregnant mice	Proportion of pregnant mice (%)	Pups/mother
LHCGR^+/N316S^ mutation mice	None	14	11	78	9 ± 1.9
	First progesterone treatment^a^	3	2	67	11 ± 0.5
	Secondary progesterone treatment^b^	1	1	100	12 ± 0
Wild type mice	None	14	14	100	14 ± 1.6

^a^Infertility mice in the None group receive the first progesterone treatment

^b^Infertility mice in the “First progesterone treatment” group receive the second progesterone treatment

## Discussion

In this study, scRNA-seq was applied to investigate the transcriptional profiles of oocytes, CGs and MGs, and then the interactions between these cell types in antral follicles. The scRNA-seq data revealed that the *Kitl*-*Kit* pathway was shown in the network. The *Kitl*-*Kit* pathway is a canonical signal pathway involved in the development of follicles [40, 42]. KIT is a multi-domain transmembrane tyrosine kinase expressed in multiple cell types, such as germ cells [49]. KITL, the ligand of KIT, is expressed in GCs and can promote oocyte growth by binding to the KIT receptor. It was reported that the interaction of KIT and KITL can activate the PI3K pathway by causing receptor dimerization and autophosphorylation [50].

The current study found that *Bmp15*, which is upstream of the *Kitl-Kit* signaling pathway, was highly expressed in oocytes. *Bmp15* was the first reported ovarian determining gene and was used as an indicator of the oocytes ability to sustain folliculogensis [51]. *Bmp15* can promote the expression of *Kitl* [52, 53] and the proliferation of GCs [54]. Another study found that reduced *Bmp15* expression led to decreased *Kitl* expression in oocytes and GCs [55]. Decreased expression of *Bmp15* and *Kitl* in follicles may increase follicle atresia and decrease ovarian follicle reserve [55]. Thus, we predicted that the BMP15-KITL-KIT-PI3K pathway was significant for oocyte development regulated by GCs.

The Kitl expressed by GCs binds to the Kit receptor and activates many downstream molecules in oocytes. Factors downstream of the BMP15-KITL-KIT-PI3K pathway include *Arf6*, which the present study showed was highly expressed in GV oocytes. Therefore, *Arf6* may be one of the key factors regulating follicular development, consistent with findings from previous studies [21, 56, 57]. Past work reported that *Arf6* is activated by cytohesin 2 in a PI3K-dependent manner during preadipocyte migration [44]. Interestingly, the present study found that cytohesin 2 was also highly expressed in GV oocytes. Past research showed that the activity of *Arf6* could be regulated by PI3K in other cell types [44]. The intra-oocyte PI3K pathway can be activated by KITL-KIT in growing oocytes [36, 40]. During follicle development, oocyte and granulosa cells coordinate intensively for the progress of follicle maturation. According to our RNA-seq data and literature, *Bmp15* is almost exclusively expressed in oocytes. *Bmp15* could trigger the expression *kitl* in granulosa cells. Then, secreted KITL binds to its receptor KIT which was expressed on oocytes and granulosa cells. After this, *Arf6* was activated in both oocytes and granulosa cells. In oocytes, BMP15-KITL-KIT-PI3K-ARF6 was supposed to regulate the cytoskeleton arrangement and meiosis. In granulosa cells, activated *Arf6* triggers the LHR signals which promote the growth and secreting the nursing materials for follicle growth. Together, the maturation of oocytes and granulosa cells is coordinated via BMP15-KITL-KIT-PI3K-ARF6 pathway during follicle development.

The present study also found that lncRNAs were highly expressed in mouse follicles, and correlated with *Bmp15*, *Kitl, Kit* and *Arf6*. This result indicates that follicular development may be regulated by lncRNAs through factors involved in the BMP15-KITL-KIT-PI3K-ARF6 pathway.

In the present study, *Arf6* was highly expressed in oocytes, CGs and MGs, and *Lhr* was highly expressed in CGs. It is well known that BMP15-KITL-KIT pathway and *Lhr* play important roles in oocytes maturation. However, whether oocyte secreted BMP15 could regulate the activity of *Lhr* on granulosa is unclear. Through single cell RNA-seq, we revealed *Arf6* is a downstream effector of BMP15-KITL-KIT pathway. Moreover, *Arf6* could regulate the internalization and signaling of LHR on granulosa [22, 58]. Therefore, this study demonstrated the links between BMP15-KITL-KIT and ARF6-LHR. To our knowledge, it is the first time to depict the important roles of BMP15-KITL-KIT/ARF6-LHR pathway in the communication between oocytes and granulosa cells during the maturation of follicles. In humans, the LHR^N312S^ mutation may inhibit oocyte maturation [27]. We established the LHR^N316S^ mutant mouse model by CRISPR/Cas9 to evaluate the impact of this LHR mutation in follicular development. For humans, it was reported that the proportion of individuals heterozygous for the LHR^N312S^ mutation was higher than those homozygous for asparagine (N/N) or serine (S/S) at this site [59]. Therefore, we chose heterozygous LHR^N316S^ mice for further study.

The numbers of corpora lutea and ovulated oocytes were lower in LHR^N316S^ mice than in control mice, although there were no differences in body weight and ovary-to-body weight ratios between the two groups. Furthermore, LHR^N316S^ females exhibited an infertility rate of 21.4% when mated with WT males. In addition, pregnant LHR^N316S^ mice ultimately produced a reduced number of offspring per litter compared with control mice. The number of oocytes from hormone-induced ovulation was also significantly lower for LHR^N316S^ compared with that for control mice. These results indicated that the LHR^N316S^ caused infertility or subfertility in mice. Similar to our findings, previous studies reported that it failed to ovulate when *LHR* was abnormal in females [25].

The levels of progesterone in LHR^N316S^ mice were significantly lower than those in control mice. Past research showed that production of progesterone by GCs during the maturation of COCs was affected by LH and follicle-stimulating hormone [60]. In porcine oocytes, progesterone decreased connexin in MGs by a progesterone receptor (PR)-mediated pathway, leading to the recovery of meiosis [61]. Therefore, it is reasonable to speculate that LHR^N316S^ may affect oocyte maturation and fertility by reducing progesterone levels.

The role of progesterone in the actions of the LHR^N316S^ mutant was investigated in vivo and in vitro. In vitro, using isolated COCs from PMSG-stimulated mice, the number of oocytes reaching metaphase II was significantly reduced for LHR^N316S^ mice compared with that for control mice. Interestingly, the oocyte maturation rate was significantly higher for progesterone-treated versus untreated cultured COCs from LHR^N316S^ mice, and was similar to that of control COCs. Then, we explored the in vivo impact of progesterone on the fertility of LHR^N316S^ mice. Initially infertile LHR^N316S^ females (identified after natural mating with WT males) all generated offspring after progesterone treatment.

Thus, these results of both in vivo and in vitro models illustrated that LHR^N316S^ affects the fertility of mice by reducing the level of progesterone. Consistent with our conclusion, reduced progesterone during the pre-ovulatory follicle stage was reported to lower pregnancy rates [62]. Conversely, a rising plasma progesterone concentration had a significant positive correlation with ovulation [63]. Simon et al*.* reported that progesterone was significant to the outcome of IVF [64]. However, there are also some opposite actions reported for progesterone. For example, progesterone inhibited follicle growth and the proliferation of GCs [65]. Higher progesterone levels were found in cows with low antral follicle counts in comparison with cows exhibiting high antral follicle counts [66]. Increased progesterone levels induced ovulation failure in crossbred Holstein heifers [67]. Progesterone alone did not have a positive effect on follicular growth [68]. Considering these diverse conclusions, we predict that the different effects of progesterone on follicular development and fertility may reflect the different actions of progesterone during distinct stages of ovary function.

## Conclusions

In conclusion, our current study detected the transcriptional profiles of oocytes, CGs and MGs by scRNA-seq and investigated the interactions between these cellular profiles. The results showed that oocytes could be regulated by CGs through the BMP15-KITL-KIT-PI3K-ARF6 signaling pathway. We generated LHR^N316S^ mutant mice by CRISPR/Cas9 technology, and revealed that LHR^N316S^ led to decreased progesterone levels, which reduced oocyte maturation and resulted in infertility or subfertility. These results also suggested that progesterone therapy may be an effective means for the clinical treatment of female infertility or subfertility.

## Supplementary Information

Below is the link to the electronic supplementary material.

Supplementary file1 Fig. S1. A flow chart for the data analysis (PDF 133 KB)

Supplementary file2 Fig. S2. Co-expression network of genes related to the regulation of oocyte development (PDF 1542 KB)

Supplementary file3 Fig. S3. Long non-coding RNAs (lncRNAs) correlated with genes involved in oocyte development (PDF 223 KB)

Supplementary file4 Fig. S4. Single cell Lhr and Arf6 expression were validated by qRT-PCR. Mann-Whitney test was used to test the differences between oocytes (O), mural granulosa cells (MG) and cumulus granulosa cells (CG). *: P < 0.05, n.s.: no significant differences (PDF 51 KB)

Supplementary file5 Fig. S5. Sanger sequencing chromatogram of genomic DNA samples from wildtype (WT) and LHRN316S mice (PDF 49 KB)

Supplementary file6 (XLSX 9 KB)

Supplementary file7 (XLS 11992 KB)

Supplementary file8 (XLS 1334 KB)

Supplementary file9 (XLSX 1212 KB)

Supplementary file10 (XLSX 2653 KB)

Supplementary file11 (XLS 307 KB)

Supplementary file12 (XLS 397 KB)

Supplementary file13 (XLS 135 KB)

Supplementary file14 (XLSX 131 KB)

Supplementary file15 (XLSX 444 KB)

Supplementary file16 (XLS 55 KB)

Supplementary file17 (XLSX 10 KB)

## Data Availability

The datasets generated and analysed during the current study are available in the NCBI Gene Expression Omnibus database [GSE241721]. https://www.ncbi.nlm.nih.gov/geo/query/acc.cgi?acc=GSE241721].

## Declarations

### Research involving animals

All procedures regarding to the animal experiment were approved by the Experimental Animal Ethics and Use Committee of Shanghai Jiao Tong University (Number: 201703004) and were conducted in line with the National Research Council Guide for Care and Use of Laboratory Animals. The ethical statement file was shown in Supplementary Material.
