# Supplementary figures and images for: Luteinizing Hormone Receptor Mutation (LHRN316S) Causes Abnormal Follicular Development Revealed by Follicle Single-Cell Analysis and CRISPR/Cas9
Source: Interdiscip Sci. 2024 Aug 16;16(4):976–89. doi: 10.1007/s12539-024-00646-7 (PMC11512921; doi:10.1007/s12539-024-00646-7)

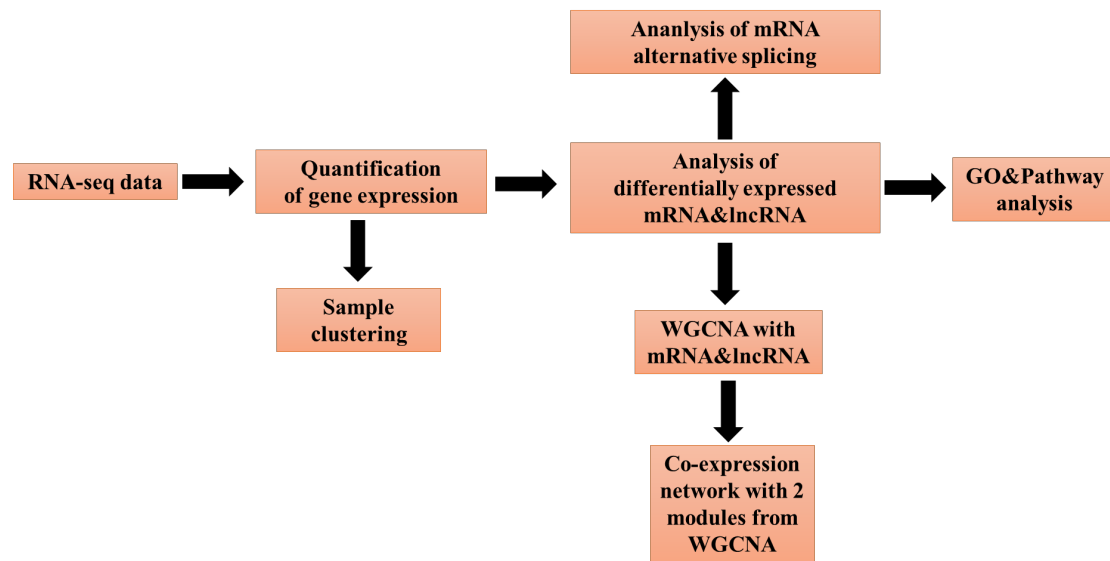

Flow chart of the RNA-seq data analysis

Supplement: Supplementary file 1 — Supplementary file1 Fig. S1. A flow chart for the data analysis (PDF 133 KB) [file 12539_2024_646_MOESM1_ESM.pdf]

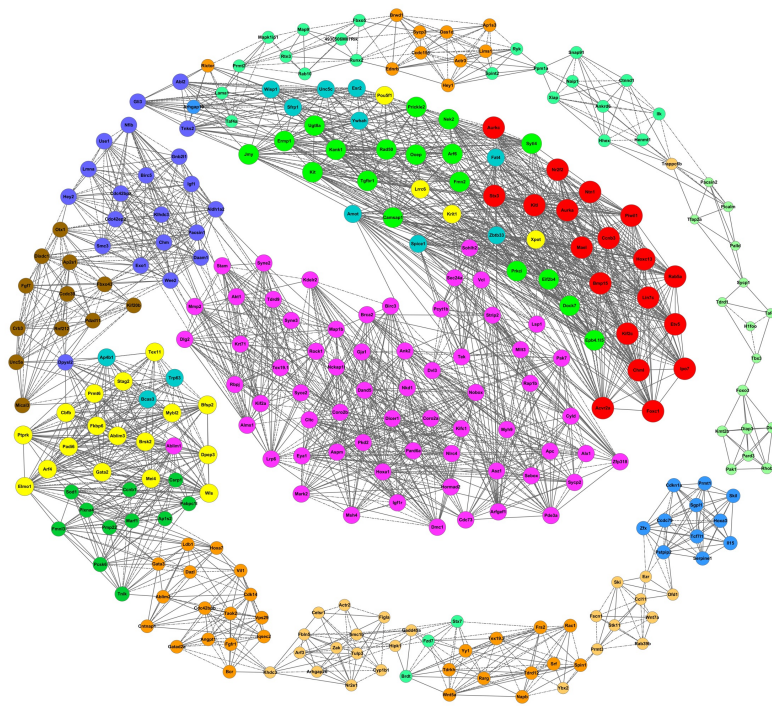

Supplement: Supplementary file 2 — Supplementary file2 Fig. S2. Co-expression network of genes related to the regulation of oocyte development (PDF 1542 KB) [file 12539_2024_646_MOESM2_ESM.pdf]

**a**

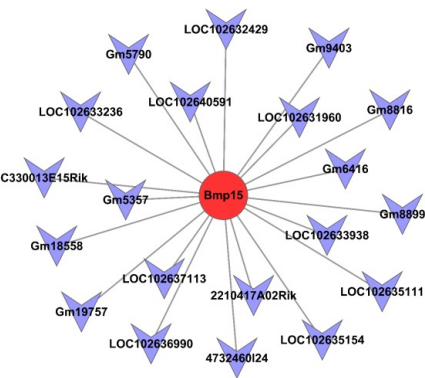

**b**

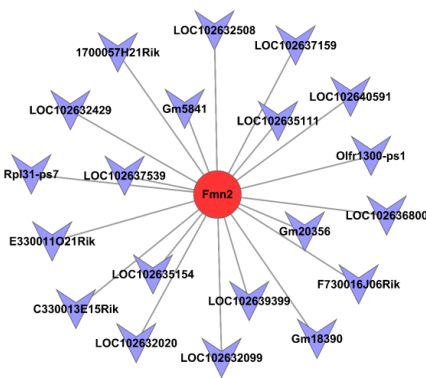

**c**

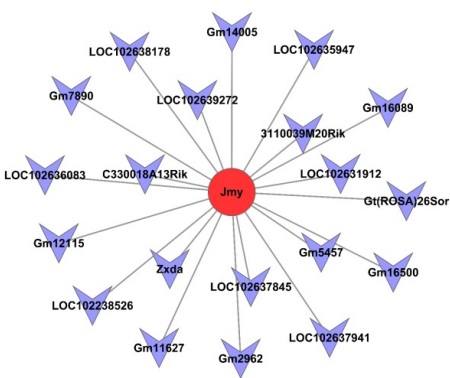

Supplement: Supplementary file 3 — Supplementary file3 Fig. S3. Long non-coding RNAs (lncRNAs) correlated with genes involved in oocyte development (PDF 223 KB) [file 12539_2024_646_MOESM3_ESM.pdf]

**a**

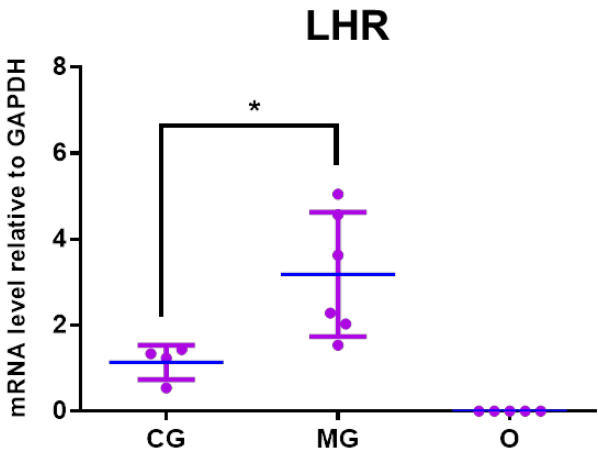

**b**

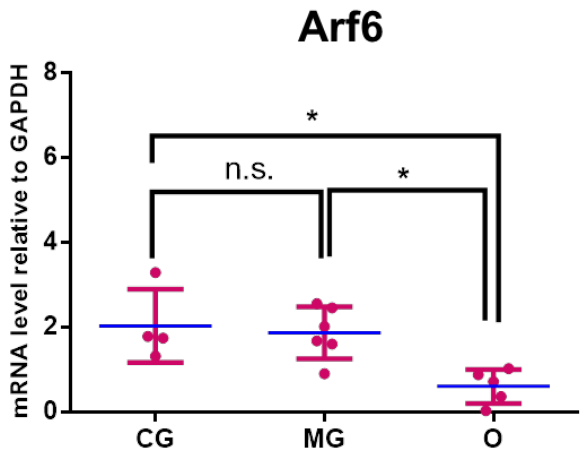

Supplement: Supplementary file 4 — Supplementary file4 Fig. S4. Single cell Lhr and Arf6 expression were validated by qRT-PCR. Mann-Whitney test was used to test the differences between oocytes (O), mural granulosa cells (MG) and cumulus granulosa cells (CG). *: P < 0.05, n.s.: no significant differences (PDF 51 KB) [file 12539_2024_646_MOESM4_ESM.pdf]

WT

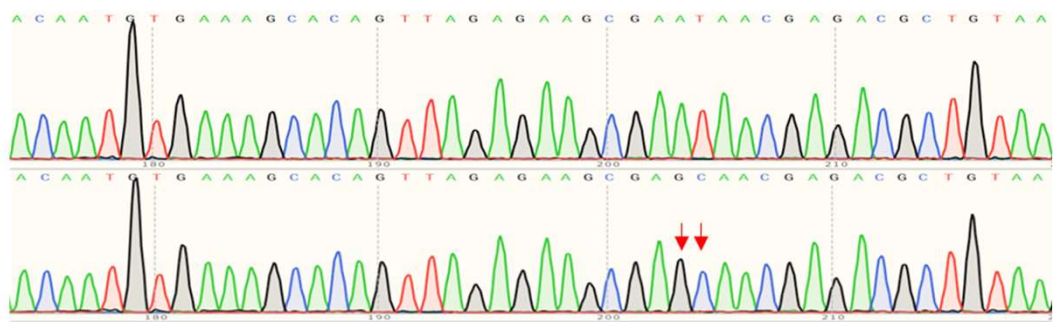

*LHR*<sup>N316S</sup>

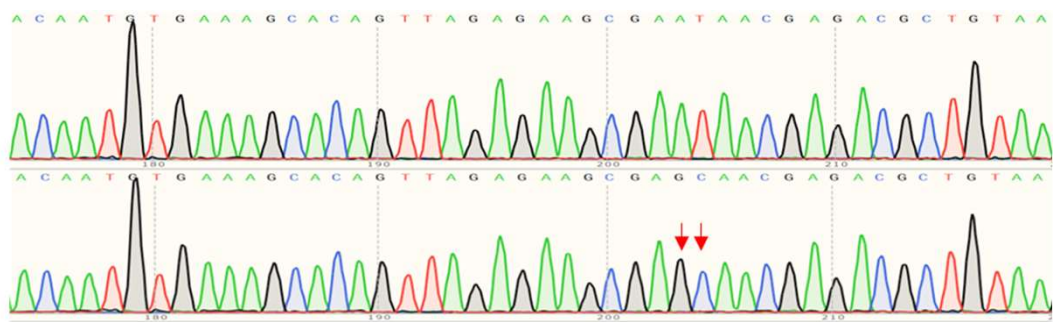

Supplement: Supplementary file 5 — Supplementary file5 Fig. S5. Sanger sequencing chromatogram of genomic DNA samples from wildtype (WT) and LHRN316S mice (PDF 49 KB) [file 12539_2024_646_MOESM5_ESM.pdf]
